# A cocktail of rapamycin, acarbose, and phenylbutyrate prevents age-related cognitive decline in mice by targeting multiple aging pathways

**DOI:** 10.1007/s11357-024-01198-w

**Published:** 2024-05-16

**Authors:** Zhou Jiang, Qianpei He, Jackson Wezeman, Martin Darvas, Warren Ladiges

**Affiliations:** 1 Department of Comparative Medicine, School of Medicine, University of Washington, Seattle, WA USA; 2 Department of Laboratory Medicine and Pathology, School of Medicine, University of Washington, Seattle, WA USA

**Keywords:** Aging pathways, Age-related cognitive impairment, Drug cocktail, Rapamycin, Acarbose, Phenylbutyrate, RNA sequencing, Immunohistochemistry, Aging mice

## Abstract

Aging is a primary risk factor for cognitive impairment and exacerbates multiple biological processes in the brain, including but not limited to nutrient sensing, insulin signaling, and histone deacetylation activity. Therefore, a pharmaceutical intervention of aging that targets distinct but overlapping pathways provides a basis for testing combinations of drugs as a cocktail. Our previous study showed that middle-aged mice treated with a cocktail of rapamycin, acarbose, and phenylbutyrate for 3 months had increased resilience to age-related cognitive decline. This finding provided the rationale to investigate the transcriptomic and molecular changes within the brains of mice that received this cocktail treatment or control treatment. Transcriptomic profiles were generated through ribonucleic acid (RNA) sequencing, and pathway analysis was performed by gene set enrichment analysis to evaluate the overall RNA message effect of the drug cocktail. Molecular endpoints representing aging pathways were measured using immunohistochemistry to further validate the attenuation of brain aging in the hippocampus of mice that received the cocktail treatment, each individual drug or control. Results showed that biological processes that enhance aging were suppressed, with an increased trend of autophagy in the brains of mice given the drug cocktail. The molecular endpoint assessments indicated that treatment with the drug cocktail was overall more effective than any of the individual drugs for relieving cognitive impairment by targeting multiple aging pathways.

## Introduction

Aging is a complex process involving the change of multifactorial pathways in all organs of the body including the brain. Aberrant progression of several critical pathways accelerates biological aging. Pathways including mammalian target of rapamycin (mTOR) signaling, insulin signaling, and histone deacetylation [1] are respectively and individually targeted by rapamycin (Rap), acarbose (Acb), and phenylbutyrate (Pba), with each drug shown to have anti-aging effects in mice [2–6]. A combination of these three drugs as a cocktail would be expected to result in a more robust outcome on preventing or slowing aging in the brain due to targeting multiple aging pathways [7].

Rap is an antibiotic clinically approved for treating organ transplant patients and neoplastic conditions [8, 9]. It has a high oral bioavailability and readily crosses the blood brain barrier. Rap blocks mTOR1, a protein shown to integrate signals from growth factors and nutrients to control protein synthesis [10]. Mechanistically, Rap downregulates mTOR1 signaling, leading to a phenocopy of caloric restriction [11]. Previous studies demonstrated that Rap feeding increased lifespan even when started late in life in both male and female mice [12, 13]. Acb is a common type 2 diabetes medication used for glucoregulatory control by delaying the digestion of complex carbohydrates. Specifically, Acb reversibly inhibits α-glucosidases within the intestinal brush border and promotes postprandial blood glucose regulation [14]. A previous study showed long-term treatment with Acb in heterogeneous background mice extended both median and maximum lifespan [4]. Pba is clinically approved as an ammonia scavenger for urea cycle disorders in children and acts as a broad inhibitor to class I and II histone deacetylases, which enhance histone acetylation and protein folding as well as decrease endoplasmic reticulum stress [15]. Extensive studies have demonstrated that increases in histone acetylation globally elevate transcription, which can be beneficial at old age as it contributes to the reversion of age-dependent decline in stress response, deoxyribonucleic acid (DNA) repair, and other genes involved in the maintenance of homeostasis [15, 16]. However, it is noteworthy that, unlike Rap and Acb, which have been shown to extend lifespan in various models, Pba did not demonstrate an increase in lifespan in mice [17].

Middle-aged mice treated for 3 months with a drug cocktail consisting of Rap, Acb, and Pba showed improvements in multiple aspects of health span including significant delays in the appearance of cognitive impairment and enhanced motor coordination [18]. In this study, we showed that mice treated with the drug cocktail had significantly better performance, with a more robust effect than any individual drug. It was therefore posited that the drug cocktail was able to retard brain aging by targeting multiple biological processes. While behavioral and physiological evidence was provided in prior studies, effects of the drug cocktail treatment on the specific processes and biological pathways have not yet been identified and evaluated. To investigate the pathways and molecular targets of brain aging in mice treated with drug cocktail or control, multivariate discriminant analysis was conducted for this study.

In order to identify age-associated pathways altered by the cocktail in the brain, transcriptomic profiles were explored through RNA sequencing. We report that the drug cocktail targeted mTOR1 signaling, insulin signaling, and histone deacetylase binding, which represent three major pathways of aging. Additional pathways of aging were also observed to be targeted by the drug cocktail including DNA damage, inflammation, senescence, and autophagy. Validation of these transcriptomic endpoints was done using immunohistochemistry to demonstrate expression levels of corresponding proteins.

## Methods

### Mouse brain tissue

**Fig. 1:**
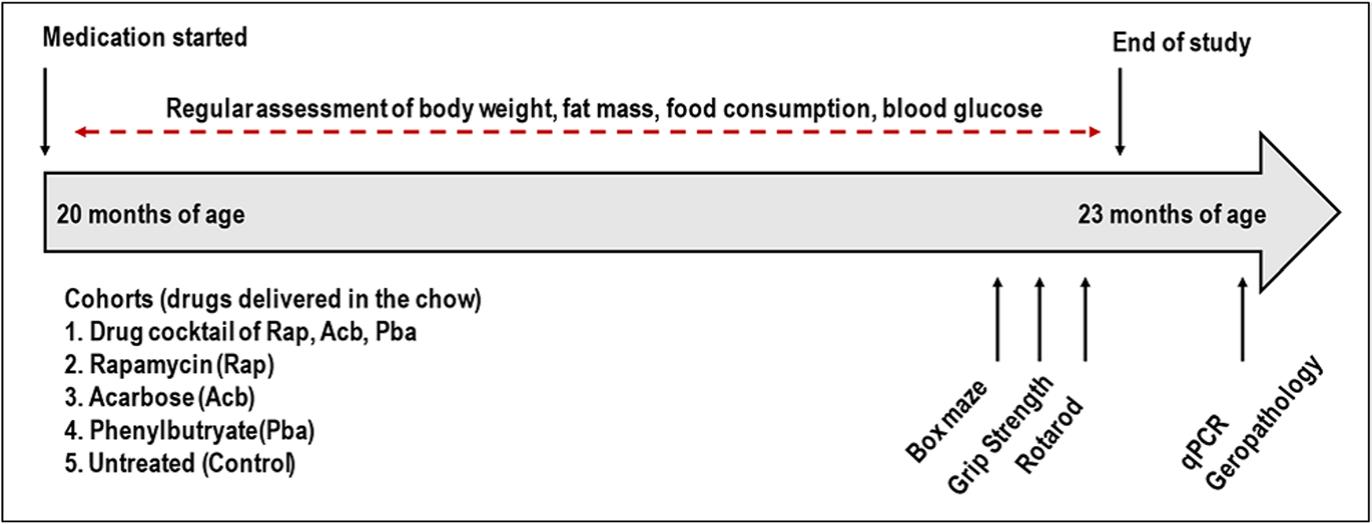
Study design for drug cocktail treatment of middle age C57BL/6J mice as previously reported (Jiang et al., 2022). Rap and Pba are orally bioavailable, and Acb acts locally in the small intestines, so all three drugs were delivered in the feed. Medicated diets were prepared by TestDiet, Inc, a division of Purina Mills (Richmond, IN). Purina 5LG food containing the test compounds, along with batches of control diet, were prepared. Microencapsulated Rap was obtained from Southwest Research Institute (San Antonio, TX) and mixed at a concentration of 14 ppm. Pba was obtained from Cayman Chemical Co., Ann Arbor, MI, and mixed at a concentration of 1000 ppm. Acb was obtained from Spectrum Chemical Mfg Corp., Gardena, CA and used at a concentration of 1000 ppm. Archived brain tissues from these mice were used in the current study

All tissues used for this study were obtained from archived brain samples of a National Institute on Aging (NIA) funded preclinical drug study, which compared aging phenotypes between the drug cocktail diet treated C57BL/6 mice with mice receiving individual drug diet or control diet [18]. In this study, both female and male mice received cocktail diet, each individual drug or placebo (control) chow. Treatment began at 20 months of age and lasted for 3 months until 23 months of age (Fig. 1). Mice treated with the drug cocktail were consistently cognitively similar to 8-month-old untreated mice compared to older mice treated with each individual drug or untreated older mice.

Nonfasted mice were euthanized via carbon dioxide asphyxiation followed by decapitation. All procedures were approved by the University of Washington IACUC as previously reported (Jiang et al., 2022). Brains were harvested and collected within 2 min of decapitation. Brains were sectioned sagittally through the midline. The parietal-temporal lobe from the left sagittal hemisphere was extracted into a sterilized vial and snap-frozen using liquid nitrogen before being transferred to a −80 °C freezer for long-term storage. The right sagittal hemisphere was fixed in 10% neutral buffered formalin (NBF) for 72 h then embedded in paraffin wax through the University of Washington Department of Comparative Medicine’s Histology Lab.

### RNA-seq

Total RNA was extracted from homogenized parietal-temporal lobe tissue of three randomly chosen drug cocktail-treated mice and three control mice using RNeasy kits (Qiagen, Redwood City CA). RNA purity was measured by Nanodrop spectrophotometer, and RIN value was obtained through 2100 Bioanalyzer. Library construction and sequencing were done through Quick Biology Inc. (Monrovia CA), using the high throughput genome sequencing platform NovaSeq 6000 with 15M paired reads. In brief, the raw RNA-seq data (SRA database PRJNA1012002) went through FASTQ quality control and cleanup and was aligned to the University of California San Diego (UCSD) C57BL/6 mice RNA data bank for further analysis.

### Quantitative reverse transcription PCR (q-PCR)

RNA preparation for q-PCR was performed in the same manner as for RNA-seq. cDNA samples were generated by OligodT (High-capacity cDNA Reverse Transcription Kit; Thermo-Fisher Cat. 4368813) reverse transcription using 5ng RNA following the manufactural standard protocol. qPCR was carried out using SYBR Green Master Mix (PowerTrack SYBR Green Master Mix, Thermo-Fisher Cat. A46109) and primer pairs. β-actine was selected as a house-keeping gene for quantification, and all q-PCR results were analyzed through the ΔΔCt method. Primer sequences are listed as follows: Cdkn2a (p16Ink4a) Fwd 5′-CCCAACGCCCCGAACT-3′, Cdkn2a (p16Ink4a) Rev 5′-GCAGAAGAGCTGCTACGTGAA-3′; β-actine Fwd 5′-CACCATTGGCAATGAGCGGTTC-3′, β-actine Rev 5′-AGGTCTTTGCGGATGTCCACGT -3′.

*Gene set enrichment analysis (GSEA*)

The DESeq2 R package [19] was used for preparing differential expression transcriptome data across all sequencing cohorts. GSEA (RRID:SCR_003199) is a useful tool to interpret high throughput expression studies and identify insights into biological pathways underlying a given phenotype [20, 21]. The GSEA algorithm scores a gene set according to how the genes in it represent increases or decreases on average in response to the regulation strength. The groups of pathways selected for GSEA analysis were predefined by previous studies [1] as major pathways of mammalian aging. Reference gene sets were extracted through Gene Ontology (GO) or Kyoto Encyclopedia of Genes and Genomes (KEGG) and Gene Ontology Molecular Function (GOMF) databases with strong validation from previous literature. For GSEA, mouse gene symbols were remapped to human gene orthologs. The GSEA analysis was done using GSEA software [20, 21] version 4.2.1, and the database was derived from the Molecular Signatures Database v5.0 [20, 22]. GSEA was based on the K-S test which weighs each observed gene within the gene set based on a given cumulative density function.

### Immunohistochemistry (IHC)

Brain tissues were randomly selected from each group. Brains from 8-month-old mice were measured for each tested endpoint as an untreated young control. Four-micrometer formalin-fixed paraffin-embedded unstained slides were used for staining. Slides were rehydrated with xylene and a series of decreasing concentrations of ethanol in water. Slides then went through 20 min of heat-mediated antigen retrieval (98 °C) in citric acid buffer (pH 6.0). Slides stained with P21 as primary antibody were immersed in ethylenediaminetetraacetic acid (EDTA) buffer (pH 8.0) for antigen retrieval. Sections were then blocked with endogenous peroxidase plus 3% H_2_O_2_, serum, avidin, and biotin blocking reagents (HRP-DAB Cell & Tissue Staining Kit, R&D Systems, Minneapolis MN) and washed in Tris-buffered saline (TBS) with 0.1% Tween 20 (TBST) in between each blocking step. Sections were then incubated in primary antibodies diluted in TBST solution overnight in a 4 °C humidified chamber. The dilution concentration for each antibody is as follows: HDAC2 (rabbit polyclonal to HDAC2, Abcam, Boston MA, Cat. ab1793, RRID: AB_305706) 1:500, MCP1 (rabbit polyclonal to MCP1, Novus Biologicals, Centennial CO, Cat. NBP1-07035, RRID: AB_1625612) 1:200, TNFα (mouse monoclonal to TNFα, Abcam, Cat. ab1793, RRID: AB_302615) 1:400, ATG5 (rabbit polyclonal anti-ATG5, Novus Biologicals, Cat. NB110-53818, RRID: AB_828587) 1:500, IL6 (rabbit polyclonal to IL-6, Abcam, Cat. ab208113, RRID: AB_2927421) 1:400, P21 (rabbit monoclonal to p21, Abcam, Cat. ab188224, RRID: AB_2734729) 1:50, and γH2AX (rabbit monoclonal to gamma H2A.X, Abcam, Cat. ab26350, RRID: AB_470861.) 1:500. Slides were rinsed thoroughly before incubating in a biotinylated secondary antibody for 30 min at room temperature. After the washing step, sections were incubated with conjugated high sensitivity streptavidin horseradish peroxidase for 30 min followed by washing steps. 3,3′-diaminobenzidine chromogen was applied to slides for 20 min until the desired intensity of staining was reached. Slides were then dehydrated and mounted for imaging and analysis.

### QuPath analysis

IHC slides were photographed under a Nikon Eclipse microscope with a Nikon D7100 camera. All photos were taken under a magnification of ×40 for capturing the entire area of the hippocampus across brain tissue slides from five randomly chosen mice per treatment group. Qupath (RRID:SCR_018257) version v2.0 was used for positive stained cell identification and optical density quantification [23]. The background staining threshold red green blue (RGB) values were calibrated consistently on sections treated with the same primary antibody regardless of specimen cohort conditions. The hippocampus was annotated with the polygon wand tool to selectively measure the staining expression within the desired region. Cellular parameters for positive staining detection were fixed based on antibody detection properties. Detected cells were calibrated and quantified by RGB-based superpixel clustering. The quantification indicated the average optical density for each detectable cell within the annotated region. An intensity heat map reflecting the staining quantification was generated for visualization purposes. Heat map gradients were adjusted to represent the intensity level across all stained slides that received the same primary antibody.

### Statistics

All numerical data analyses were conducted in Prism (GraphPad Software, La Jolla CA, RRID:SCR_002798). Two-way analysis of variance (ANOVA) followed by Tukey’s post hoc test was used to compare the population difference between cohorts. *p*-value ≤ 0.05 was considered statistically significant and presented as mean ± standard of mean (SEM). For GSEA, lists of ranked genes based on a score calculated by the K-S test as −log_10_ of *p*-value multiplied by fold-change, either upregulated or downregulated. Enrichment score (ES) was calculated by the difference between 0 and the extremes of the entire ranked gene list. The normalized enrichment scores reflected default parameters with ES over the average of positive to negative extremes within the gene set [20].

## Results

### The brains from mice treated with a cocktail of Rap, Acb, and Pba showed downregulation of transcriptomic profiles targeted by each individual drug

**Fig. 2:**
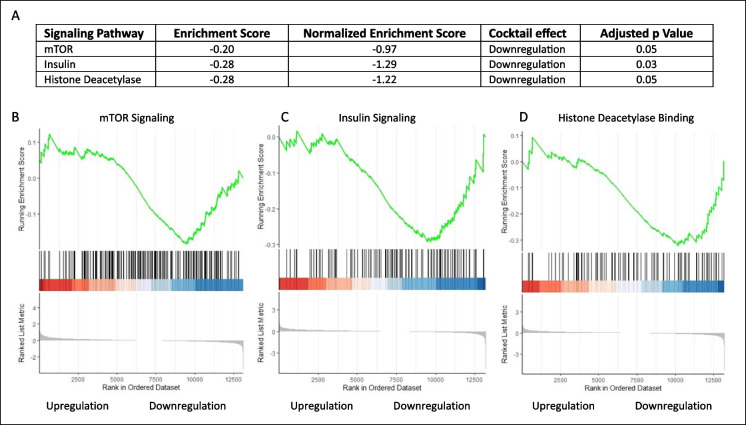
GSEA enrichment scores of brain tissues from mice treated with drug cocktail compared to brains from untreated mice (control). The drug cocktail downregulated mTOR1 signaling, insulin signaling, and histone deacetylase binding. **A** Table summarizing the enrichment score data. **B** mTOR1 signaling pathway, **C** insulin signaling pathway, and **D** histone deacetylase binding pathway. *p* ≤ 0.05. *N* = 3/cohort

The parietal-temporal lobe with hippocampus from mice treated with cocktail or control chow was randomly selected for RNA-seq. Total RNA that was purified from homogenized tissue with hippocampus was utilized. Three pathways associated with each individual drug were suppressed significantly in the brains of mice that received the drug cocktail (Fig. 2).

The Rap-medicated reduction on mTOR1 signaling was reflected by the enhanced expression of downregulating genes. As an anabolic signaling kinase, active mTOR1 translates to rapid cell growth and proliferation. Emerging studies support the idea that lower mTOR1 activity leads to decreased nutrient signaling and extends longevity [24]. The overall insulin signaling referenced to the KEGG database was negatively regulated by the cocktail through enrichment of downregulating or suppressing genes. Insulin signaling is one of the most conserved aging-controlling pathways in evolution [24–26]. In addition to the beneficiary effect from Rap, Acb promotes glucoregulatory control and improves glucose homeostasis and insulin sensitivity. Current evidence suggests that interventions that constitutively decreased insulin signaling may prevent or reverse the adverse effects associated with aging [27]. Here, induced downstream effectors were seen in the histone deacetylase binding pathway for cocktail-treated mice. Pba targets epigenetic function by inhibiting histone deacetylation, which silences genes that play a protective role in maintaining systemic as well as neuronal function. Downregulated histone deacetylation alters accessibility of chromatin and allows DNA-binding proteins to interact with exposed sites to activate gene transcription and downstream cellular function [28, 29].

### Altered regulation of transcriptomic profiles representing multiple pathways of aging was observed in the brains of mice treated with the drug cocktail

**Fig. 3:**
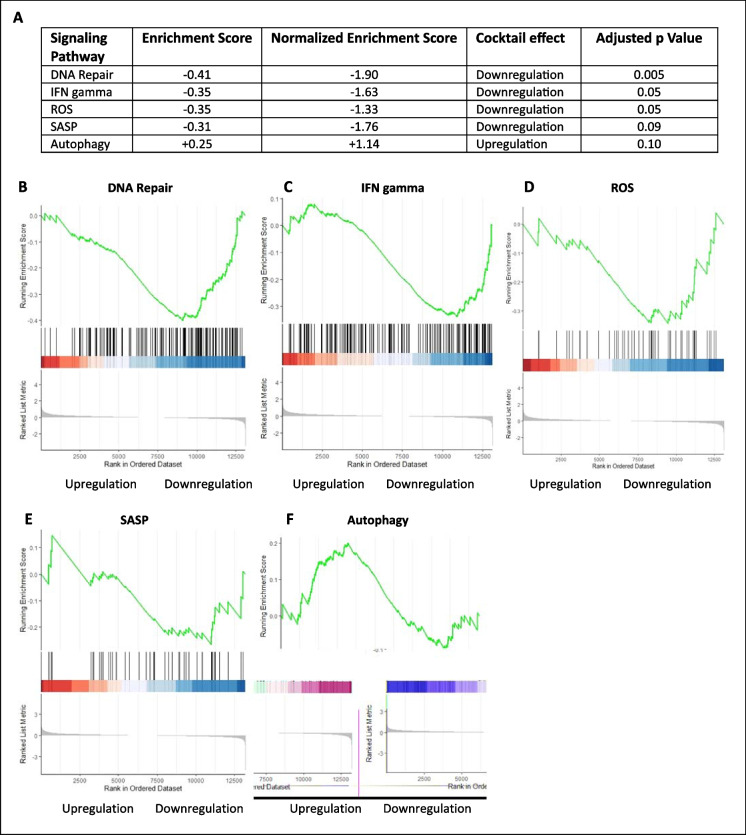
GSEA enrichment scores of aging pathways in brain tissues from mice treated with drug cocktail compared to brains from untreated mice (control). **A** Table summarizing the enrichment score data. **B** Genes involved in DNA repair. **C** Genes related to secretion of interferon-γ. **D** Genes related to ROS (reactive oxygen species) pathway. **E** Genes included for SASP (senescence-associated secretory phenotype). **F** Genes related to autophagy. *p* ≤ 0.05 except for SASP and autophagy, which were trending but not significant. *N* = 3/group

Five pathways associated with each individual drug were altered in the brains of mice that received the drug cocktail (Fig. 3A). Brains from treated mice showed a downregulated pattern of DNA repair, suggesting a reduced tendency for DNA damage (Fig. 3B). The DNA repair pathway from the GO database includes both up- and down-stream genes involved with DNA repair that makes this an ideal illustration for addressing the level of DNA damage [30]. The innate immune sensing pathway with interferon-γ as a downstream effector was chosen to represent primary inflammatory response in the brain [31]. This pathway was downregulated in the brains of mice treated with the drug cocktail (Fig. 3C).

Normal cell division is halted when cellular senescence occurs. As senescence accumulates with increasing age, an elevated expression of both systemic and local senescence-associated secretory phenotype (SASP) is observed in neuronal cells [32]. The production of reactive oxygen species (ROS) often promotes cells into senescent phase [33]. ROS and SASP pathways are commonly designated to measure upstream and downstream transcriptional targets of senescence. Both pathways were downregulated in the brains of mice treated with the cocktail. However, only the ROS pathway was significant (Fig. 3D, E). The GSEA analysis for SASP was referenced with the exclusive Reactome gene database which led to fewer genes presented within the cluster causing fewer hits from the testing groups. Even with a nominal *p*-value greater than 0.05, the enrichment plot exhibited a heavily skewed downregulating trend with a distinctive normalized enrichment score of −1.76.

The GSEA data in our study indicated an upregulated trend for autophagy with data skewed upwards for mice receiving the drug cocktail (Fig. 3F). However, the nominal *p*-value was not significant at the 0.05 level. Genomic heat map interpretation suggested that the K-S ranking for the autophagy pathway may be affected by internal variance within the treatment cohort.

### Protein expression levels of aging pathways corresponded to transcriptomic profiles in brains from mice treated with the drug cocktail

**Fig. 4:**
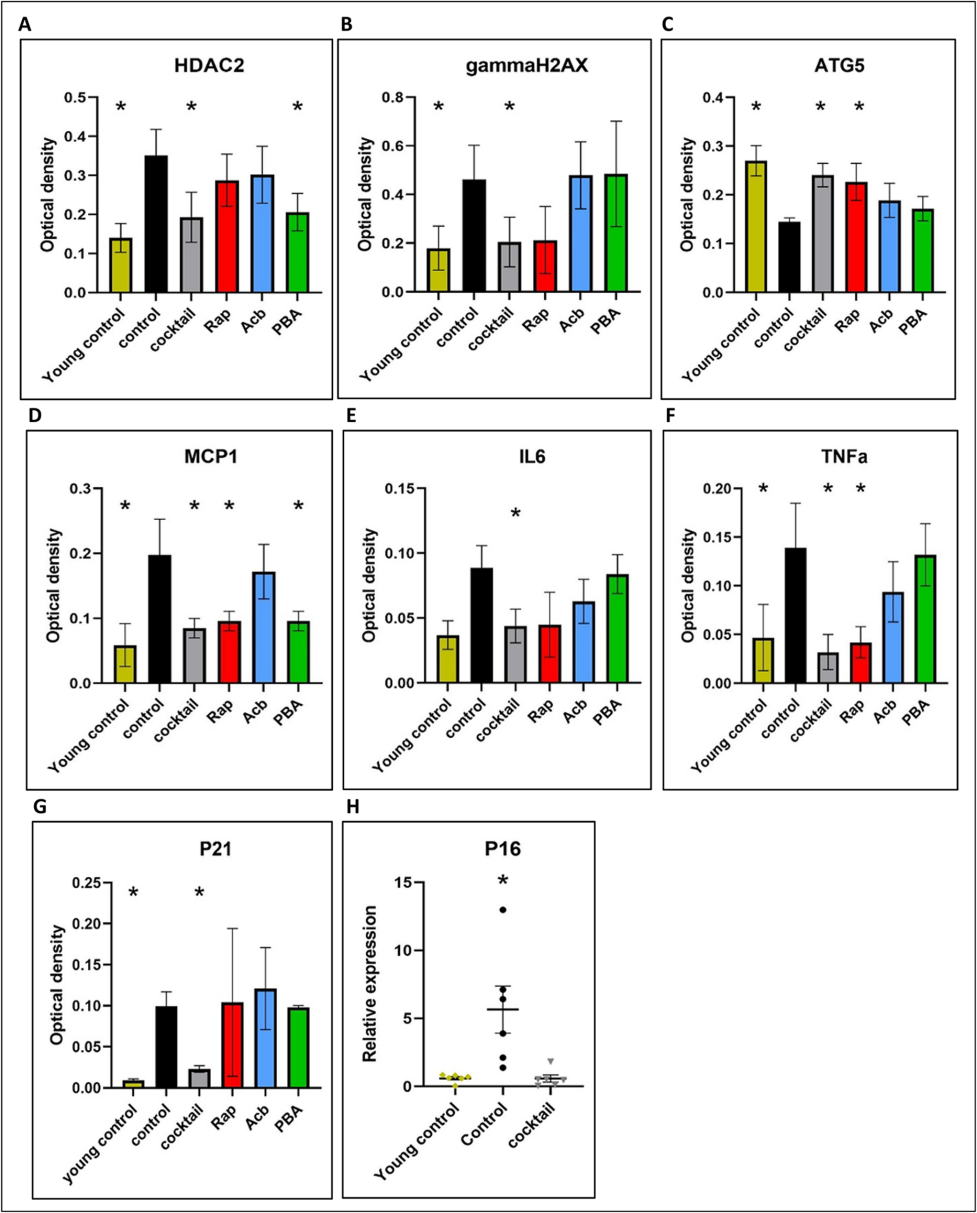
QuPath generated optical density values with ± SEM of all positive stained cells in the hippocampus for each tested antibody. The cocktail treatment group and individual drug treatment groups were compared to a 23-month-old untreated group (control) and an 8-month-old untreated group (young control). **A** HAC2; **B** γH2AX; **C** ATG5; **D** MCP-1; **E** IL-6; **F** TNF-alpha; **G** P21. *p ≤ 0.05 by two-way ANOVA followed by the Tukey post hoc test across each treatment condition. **H** q-PCR value for P16. *p ≤ 0.05 using one-way ANOVA followed by the Tukey post hoc test. *N* = 6/cohort for all tests

Images of IHC stains were digitally processed through Qupath for heat map demonstration and optical density quantification. The intensity results showed that brains from cocktail-treated mice had significantly less expression of γH2AX and HDAC2 while Pba only suppressed the expression of HDAC2 (Fig. 4A, B). HDAC2 is a class I histone deacetylation protein that participates in the DNA damage response as it is rapidly recruited to DNA damage sites to promote hypo-acetylation [34]. γH2AX refers to the X isoform of the histone H2A which resides closely with DNA as a part of the nucleosome. Phosphorylation of γH2AX is an early repair response to the detection of double-strand breaks or replication stress. The accumulation of HDAC2 and γH2AX in brain tissue has often been referred to as a surrogate marker of DNA damage [35].

Reduced levels of all three inflammatory molecules were observed for mice treated with the drug cocktail. Rap alone reduced the level of MCP-1 and TNF-alpha as Pba alone only reduced MCP-1 (Fig. 4C–E). General inflammation can be assessed by MCP-1, a pro-inflammatory cytokine that selectively recruits glial cells in the CNS [36]. IL-6 is secreted by parenchymal macrophages in the CNS and is critical for the progression of inflammatory disorders of neural tissue [37]. TNF-alpha is a pro-adhesive inflammatory factor that upregulates NF-κB. Increased activation of TNF-alpha induces both acute and chronic neuroinflammation [31]. Notably, treatment with Rap and Pba altered the expression of most of the tested biomarkers independently. However, neither of these two treatments affected the expression of P21, a marker associated with cellular senescence (Fig. 4G). The combination treatment uniquely downregulated P21 expression, indicating a synergistic or additive effect that is not replicated by any individual compound.

Biomarkers for senescence were decreased in the brain of cocktail-treated mice, and no effect was observed in other treatment cohorts (Fig. 4F, G). P16 and P21 are endpoints for identifying senescence phenotypes in murine models [36]. P21 is a downstream effector of P53 transcription factor and acts as a primary mediator for downstream cell cycle arrest. P16 is essential for oncogene-induced senescence and a cyclin-dependent kinase inhibitor that slows cell division by preventing the progression of the cell cycle from the G1 phase to the S phase [1]. P16 was measured through q-PCR due to the lack of validated commercially available antibody for IHC in the mouse brain. ATG5, a common biomarker for measuring autophagy, binds with beclin and promotes macroautophagy induction [38]. ATG5 levels were increased in the brain tissue of both cocktail and Rap-treated mice (Fig. 4H).

**Fig. 5:**
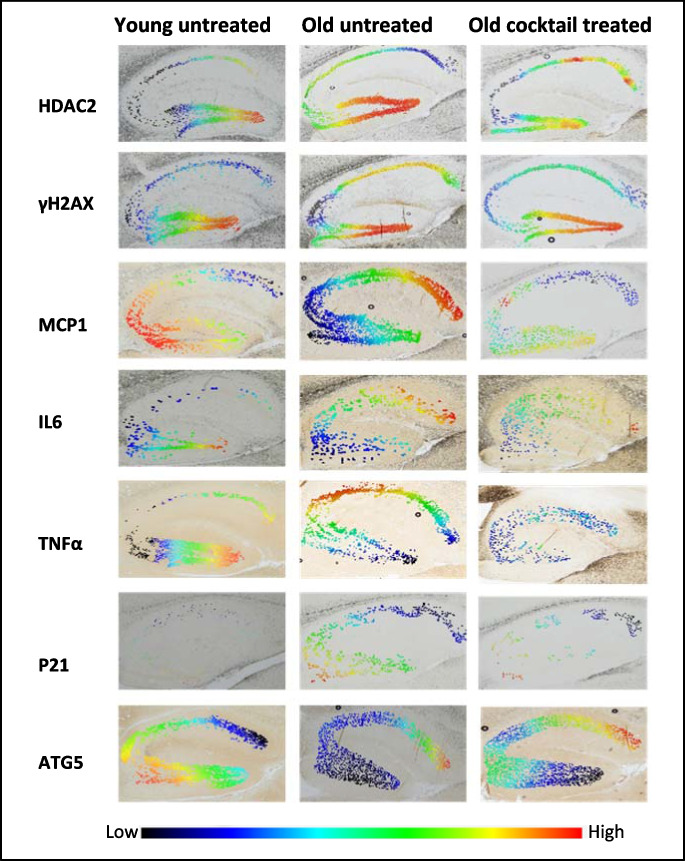
Optical density heatmaps from hippocampal sections generated using QuPath digital imaging. Each row represents staining groups of selected antibodies while each column indicates treatment cohorts. Brains from 8-month-old young control mice and 23-month-old mice are listed for negative and positive control demonstration, respectively. Each image presented is representative of typical findings from single mouse brain tissue slides, randomly selected from multiple individual samples per treatment group. ×40 magnification

Heatmaps based on regional optical density provided additional insight on the distribution of staining within the hippocampus region. While five mouse samples were analyzed per treatment group, these IHC images presented (Fig. 5) were randomly selected from a single mouse representative of each treatment group to visually exemplify typical findings. Staining intensity was visualized by a color gradient, where red represents high levels and blue represents low levels (Fig. 5). The heatmap paralleled the quantitative data generated from positive superpixel analysis.

HDAC2 and γH2AX, protein markers associated with DNA damage and repair, observed a stark contrast in expression levels between the young untreated mice, which show minimal expression indicative of less DNA damage, and the old untreated mice, which display heightened expression levels as denoted by warmer colors, suggesting increased DNA damage or repair activity characteristic of aging. However, in the old cocktail-treated group, there is a notable shift towards the cooler spectrum for γH2AX, aligning with the expression patterns observed in the young untreated mice, hinting at the treatment’s efficacy in mitigating DNA damage. Similarly, the effect of treatment is also visible in HDAC2, affecting different regions within the hippocampus.

In terms of the inflammatory markers MCP1, IL6, and TNFα, the young untreated mice exhibit relatively low levels of these markers, whereas old untreated mice show an expected increase, consistent with an age-related pro-inflammatory state. Intriguingly, the old mice that received the cocktail treatment exhibit much lower expression of these inflammatory markers, closely resembling the young untreated profile, which suggests a substantial anti-inflammatory effect of the cocktail treatment.

When examining senescence marker P21 and autophagy-associated marker ATG5, we see a low expression of P21 in young untreated mice and a drastic increase in the old untreated mice. The old cocktail-treated mice, however, show reduced P21 expression, implying that the treatment might be influencing cellular mechanisms to retard senescence. Meanwhile, ATG5 levels, which are moderately expressed in young untreated mice and decline with age, seem to be restored in the old cocktail-treated mice, indicating a rejuvenation of autophagic processes often compromised during aging.

## Discussion

Age-related cognitive decline in the brain is characterized by the progressive loss of physiological integrity. This deterioration can lead to early stages of neurodegenerative conditions such as Alzheimer’s disease and other dementias, affecting millions of older people [39]. Currently, aging is a subject of scientific scrutiny involving the aggravation of multiple different, but interconnected biological processes. Targeting multiple factors that contribute to the progression of aging will likely provide a more robust attenuation of age-related cognitive dysfunction than any single drug [7]. Significant improvement of cognitive performance was observed in middle-aged mice treated for 3 months with a drug cocktail composed of Rap, Acb, and Pba [18]. Selection of these specific drugs for a multiplex approach was based on their well-established roles in promoting health span and prolonging lifespan in mice [4, 11, 15]. Phenotypic improvement observed from this preclinical study suggested the drug cocktail may be targeting multiple pathways associated with aging.

The comparison of mechanistic pathways in the brains from mice receiving drug cocktail versus control was evaluated through GSEA on RNA-seq data. The results showed that the drug combination alleviated the aberrant progression of age-associated pathways not only directly targeted by each drug but also extensively relevant to overall cocktail effects. Molecular endpoints that manifested among tested aging pathways were further analyzed between all treatment cohorts. Quantification of IHC digital imaging provided additional evidence that the cocktail treatment showed a more comprehensive effect on ameliorating common aspects of aging compared to the administration of each individual drug in the cocktail.

A set of pathways has been considered to characterize the process of aging. Several of these pathways are involved in dysregulated nutrient sensing, enhanced insulin signaling intensity, and epigenetic alterations. Activation of mTOR1 signaling, insulin signaling, and histone deacetylase binding pathways can easily antagonize these three biological processes. Emerging studies suggest that Rap, Acb, and Pba can directly target these pathways in various mammalian models [7]. Our results indicated that all three pathways were downregulated in the brain of cocktail-treated mice at the transcriptomic level. Furthermore, cocktail treatment suppressed the aggravation of several other common aging hallmarks including DNA damage, inflammation, and senescence. DNA damage caused by changes to chromatin organization can be mediated through increasing acetylation of histones, suggesting Pba may decrease DNA double-strand breaks or replication stress [40]. mTOR implicates genome integrity maintenance, and it has been demonstrated that active mTOR1 expression interplays with DNA damage response systems and promotes growth and survival for cells undergoing constitutive DNA damage [30]. This mechanism aligned with our observation that mice with suppressed mTOR1 signaling and histone deacetylase binding also showed decreased DNA damage level.

mTOR1 is one of the key regulators for chronic inflammatory response as it controls inflammation-associated cell proliferation and migration. Rap as an anti-proliferative mTOR1 inhibitor was proven to prevent endothelial cell overgrowth and is considered a potential reagent for suppressing the primary immune response [41]. Acb was observed to lower glucose excursions and interfere with neuro-inflammatory reactions [42]. This provides the rationale for both Rap and Acb contributing to a lower level of inflammation. Rap was found to suppress the downstream complexes of mTOR1 signaling including NF-κB [43], STAT3 [44], and Akt signaling [45], hence reducing cellular senescence [1, 46]. Increasing evidence implicates cellular senescence in tissue is associated with dysregulated insulin regulation [47], which suggests that Acb may contribute to reducing the senescence-associated inflammatory response. Moreover, DNA damage is parallel to the production of senescence-associated inflammatory cytokines [15], which hints to a potential senescence-suppressing effect from the administration of Pba.

Autophagy, one of the main activities of the principal proteolytic system implicated in protein quality control, declines with aging. Rap can restore the autophagic flux and enhance autophagy by restoring mitochondrial and lysosomal functions [48]. The phenocopy of caloric restriction from Rap and antihyperglycemic effect from Acb are also known to increase autophagy activation [1]. Interestingly, a strong trend of upregulated autophagy was observed in the brains of mice that received the cocktail.

IHC assessment of age-related molecular endpoints provides insight into how drug treatment can effectively target the expression of specific biomarkers within the brain. Data from this study showed a consistently significant alleviation of expression levels in HDAC2, γH2AX, MCP-1, IL-6, TNF-alpha, and P21 from mice treated with the cocktail compared to expression levels in control mice. The results shed light on the impact of each individual drug on these molecular endpoints. Specifically, Rap was shown to significantly alter MCP-1, TNF-alpha, and ATG5 levels, whereas Pba was shown to decrease HDAC2 and MCP-1 levels. Acb alone was not associated with a reduction in any of the targeted markers, yet its inclusion in the cocktail appears to have enhanced the overall efficacy of the cocktail.

A notable observation was the unique modulation of P21 by the cocktail, absent in the monotherapies, suggesting that the combined drugs engage additional biological pathways not influenced by rapamycin or phenylbutyrate alone. This effect could result from a synergistic interaction, where the collective action of the drugs is greater than the sum of their individual effects. These findings imply that the cocktail has a synergistic advantage, offering a more comprehensive approach to modulating age-related biomarkers than any single agent.

All three drugs involved in the cocktail are Food and Drug Administration (FDA)—approved for clinical conditions in people and considered safe with little or no adverse effects. A geropathology assessment in mice from a previous preclinical study using this drug combination showed that the cocktail treatment decreased age-related lesions in all major organs without showing any histology findings related to drug toxicity or abnormal clinical pathology [18].

**Fig. 6:**
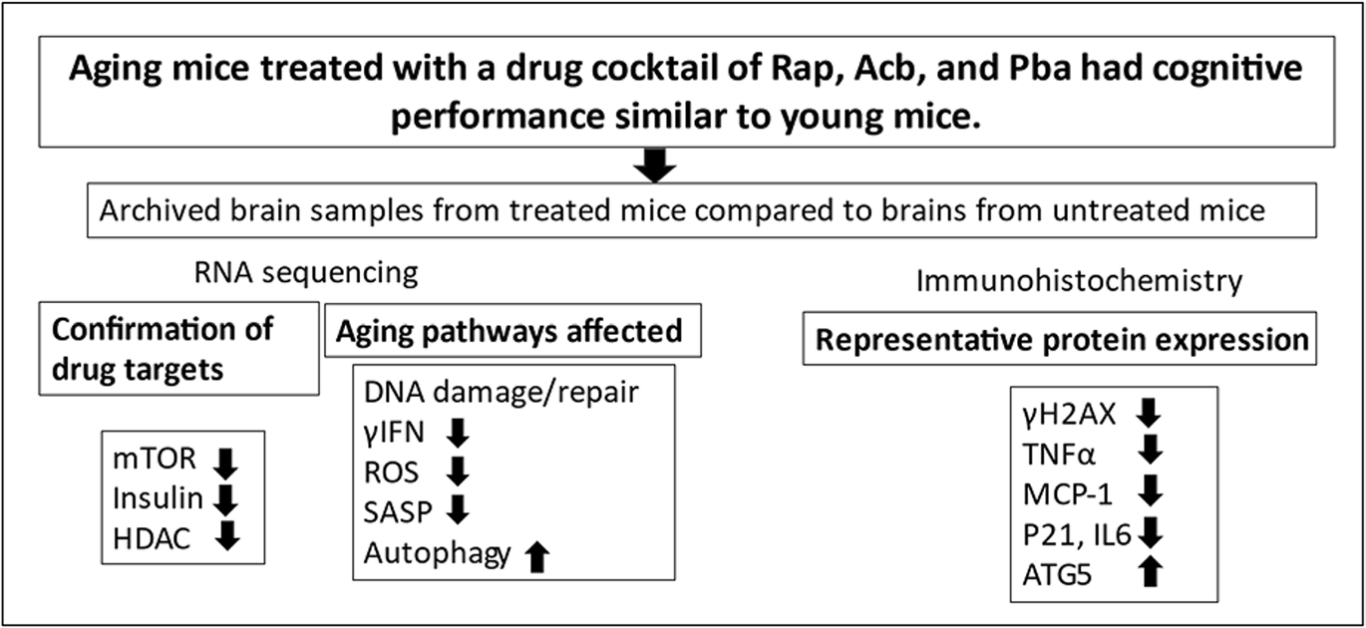
Summary of the main findings reported in this study. Abbreviations: HDAC, histone deacetylase; γIFN, gamma interferon; ROS, reactive oxygen species; SASP, senescence-associated secretory phenotype; TNFα, tumor necrosis factor alpha; ATG5, autophagy-related 5; downward arrow = downregulation; upward arrow = upregulation

In conclusion, the framework demonstrated in our study of mapping the alleviating effects of a cocktail of Rap, Acb, and Pba, applied to aging-associated pathways and molecular endpoints, provided a cross-sectional perspective of associated changes in the brains of middle-aged mice (Fig. 6). The observations suggest the cocktail is an encouraging blueprint for designing future drug combinations for preventing, reversing, or delaying age-related cognitive impairment and decreasing the risk for the development of more severe degenerative conditions such as Alzheimer’s disease.

## Data Availability

All RNA-sequencing data has been uploaded to the SRA database. It will be released upon the publication of the manuscript. The reference number is PRJNA1012002.

## References

[1] López-Otín C, Blasco MA, Partridge L, Serrano M, Kroemer G. Hallmarks of aging: an expanding universe. Cell. 2023;186(2):243–78. 10.1016/j.cell.2022.11.001. 36599349 10.1016/j.cell.2022.11.001

[2] Lesniewski, L.A., Seals, D.R., Walker, A.E., Henson, G.D., Blimline, M., Trott, D.W., Bosshardt, G.C., LaRocca, T.J., Lawson, B.R., Zigler, M.C. and Donato, A.J. Dietary rapamycin supplementation reverses age-related vascular dysfunction and oxidative stress, while modulating nutrient-sensing, cell cycle, and senescence pathways. (2017); 16(1):17–26. 10.1111/acel.12524 10.1111/acel.12524 PMC5242306 27660040

[3] Lin A-L, Jahrling JB, Zhang W, DeRosa N, Bakshi V, Romero P, Galvan V, Richardson A. Rapamycin rescues vascular, metabolic and learning deficits in apolipoprotein E4 transgenic mice with pre-symptomatic Alzheimer’s disease. J Cereb Blood Flow Metab. 2016;37(1):217–26. 10.1177/0271678x15621575. 10.1177/0271678x15621575 PMC5167110 26721390

[4] Harrison DE, Strong R, Alavez S, Astle CM, DiGiovanni J, Fernandez E, Flurkey K, Garratt M, Gelfond JAL, Javors MA, Levi M, Lithgow GJ, Macchiarini F, Nelson JF, Sukoff Rizzo SJ, Slaga TJ, Stearns T, Wilkinson JE, Miller RA. Acarbose improves health and lifespan in aging HET3 mice. Aging Cell. 2019;18(2):e12898. 10.1111/acel.12898. 30688027 10.1111/acel.12898 PMC6413665

[5] Harrison DE, Strong R, Allison DB, Ames BN, Astle CM, Atamna H, Fernandez E, Flurkey K, Javors MA, Nadon NL, Nelson JF, Pletcher S, Simpkins JW, Smith D, Wilkinson JE, Miller RA. Acarbose, 17-α-estradiol, and nordihydroguaiaretic acid extend mouse lifespan preferentially in males. Aging Cell. 2013;13(2):273–82. 10.1111/acel.12170. 24245565 10.1111/acel.12170 PMC3954939

[6] Carvajal‐Flores FN, Díaz A, Flores‐Gómez GD, Cruz F, Flores G. Phenylbutyrate ameliorates prefrontal cortex, hippocampus, and nucleus accumbens neural atrophy as well as synaptophysin and GFAP stress in aging mice. Synapse. 2020.10.1002/syn.22177 10.1002/syn.22177 32531811

[7] Sharma K, Wang J, Jiang Z, Klug J, Darvas M, Imai DM, Snider TA, Niedernhofer LJ, Ladiges W. The rationale for testing drug combinations in aging intervention studies. Aging Pathobiol Ther. 2019;1(1):01–4. 10.31491/apt.2019.12.001. 10.31491/apt.2019.12.001

[8] Cowan PA, Heizer KE. Sirolimus: mammalian target of rapamycin inhibitor to prevent kidney rejection. Nephrol Nurs J. 2000;27(6):623–5. 16649344

[9] Touzot M, Soulillou JP, Dantal J. Mechanistic target of rapamycin inhibitors in solid organ transplantation. Curr Opin Organ Tran. 2012;17(6):626–33. 10.1097/mot.0b013e32835a4be2. 10.1097/mot.0b013e32835a4be2 23080066

[10] Johnson SC, Rabinovitch PS, Kaeberlein M. mTOR is a key modulator of ageing and age-related disease. Nature. 2013; 493 7432 338–45 10.1038/nature11861 10.1038/nature11861 PMC3687363 23325216

[11] Miller RA, Harrison DE, Astle CM, Fernandez E, Flurkey K, Han M, Javors MA, Li X, Nadon NL, Nelson JF, Pletcher S, Salmon AB, Sharp ZD, Van Roekel S, Winkleman L, Strong R. Rapamycin-mediated lifespan increase in mice is dose and sex dependent and metabolically distinct from dietary restriction. Aging Cell. 2014;13(3):468–77. 10.1111/acel.12194. 24341993 10.1111/acel.12194 PMC4032600

[12] Harrison DE, Strong R, Sharp ZD, Nelson JF, Astle CM, Flurkey K, Nadon NL, Wilkinson JE, Frenkel K, Carter CS, Pahor M, Javors MA, Fernandez E, Miller RA. Rapamycin fed late in life extends lifespan in genetically heterogeneous mice. Nature. 2009;460(7253):392–5. 10.1038/nature08221. 19587680 10.1038/nature08221 PMC2786175

[13] Strong R, Miller RA, Bogue M, Fernandez E, Javors MA, Libert S, Marinez PA, Murphy MP, Musi N, Nelson JF, Petrascheck M, Reifsnyder P, Richardson A, Salmon AB, Macchiarini F, Harrison DE. Rapamycin‐mediated mouse lifespan extension: late‐life dosage regimes with sex‐specific effects. Aging Cell. 2020; 19(11). 10.1111/acel.13269 10.1111/acel.13269 PMC7681050 33145977

[14] Gibbs VK, Brewer RA, Miyasaki ND, Patki Amit, Smith DL. Sex-dependent differences in liver and gut metabolomic profiles with acarbose and calorie restriction in C57BL/6 mice. J Gerontol. 2017;73(2):157–65. 10.1093/gerona/glx127. 10.1093/gerona/glx127 PMC5861978 28651373

[15] Wiley JC, Meabon JS, Frankowski H, Smith EA, Schecterson LC, Bothwell M, Ladiges WC. Phenylbutyric acid rescues endoplasmic reticulum stress-induced suppression of APP proteolysis and prevents apoptosis in neuronal cells PloS One. 2010; 5(2):e9135. 10.1371/journal.pone.0009135 10.1371/journal.pone.0009135 PMC2817752 20161760

[16] Pasyukova EG, Vaiserman AM. HDAC inhibitors: a new promising drug class in anti-aging research. Mech Ageing Dev. 2017;166:6–15. 10.1016/j.mad.2017.08.008. 28843433 10.1016/j.mad.2017.08.008

[17] Harrison DE, Strong R, Reifsnyder P, Rosenthal N, Korstanje R, Fernandez E, Flurkey K, Ginsburg BC, Murrell MD, Javors MA, Lopez-Cruzan M, Nelson JF, Willcox BJ, Allsopp R, Watumull DM, Watumull DG, Cortopassi G, Kirkland JL, Tchkonia T, Choi YG, Yousefzadeh MJ, Robbins PD, Mitchell JR, Acar M, Sarnoski EA, Bene MR, Salmon A, Kumar N, Miller RA. Astaxanthin and meclizine extend lifespan in UM-HET3 male mice; fisetin, SG1002 hydrogen sulfide donor, dimethyl fumarate, mycophenolic acid, and 4-phenylbutyrate do not significantly affect lifespan in either sex at the doses and schedules used. Geroscience. 2024; 46(1):795-816. 10.1007/s11357-023-01011-0 10.1007/s11357-023-01011-0 PMC10828146 38041783

[18] Jiang Z, Wang J, Imai D, Snider T, Klug J, Mangalindan R, et al. Short term treatment with a cocktail of rapamycin, acarbose and phenylbutyrate delays aging phenotypes in mice. Sci Rep. 2022;12(1):7300. 35508491 10.1038/s41598-022-11229-1 PMC9067553

[19] Love MI, Huber W, Anders S. Moderated estimation of fold change and dispersion for RNA-seq data with DESeq2. Genome Biol. 2014; 15(12). 10.1186/s13059-014-0550-8 10.1186/s13059-014-0550-8 PMC4302049 25516281

[20] Subramanian A, Tamayo P, Mootha VK, Mukherjee S, Ebert BL, Gillette MA, et al. Gene set enrichment analysis: a knowledge-based approach for interpreting genome-wide expression profiles. Proc Natl Acad Sci USA. 2005;102(43):15545–50. 16199517 10.1073/pnas.0506580102 PMC1239896

[21] Mootha VK, Lindgren CM, Eriksson K-F, Subramanian A, Sihag S, Lehar J, Puigserver P, Carlsson E, Ridderstråle M, Laurila E, Houstis N, Daly MJ, Patterson N, Mesirov JP, Golub TR, Tamayo P, Spiegelman B, Lander ES, Hirschhorn JN, Altshuler D. PGC-1α-responsive genes involved in oxidative phosphorylation are coordinately downregulated in human diabetes. Nat Genet. 2003;34(3):267–73. 10.1038/ng1180. 12808457 10.1038/ng1180

[22] Liberzon A, Birger C, Thorvaldsdóttir H, Ghandi M, Mesirov Jill P, Tamayo P. The molecular signatures database hallmark gene set collection. Cell Systems. 2015;1(6):417–25. 10.1016/j.cels.2015.12.004. 26771021 10.1016/j.cels.2015.12.004 PMC4707969

[23] Lee A, Jiang Z, Zhu L, Ladiges W. QuPath. A new digital imaging tool for geropathology. Aging Pathobiology and Therapeutics. 2020;2(2):114–6. 10.31491/apt.2020.06.024 35083445 10.31491/apt.2020.06.024 PMC8789032

[24] Fontana L, Partridge L, Longo VD. Extending healthy life span–from yeast to humans. Science. 2010;328(5976):321–6. 10.1126/science.1172539. 20395504 10.1126/science.1172539 PMC3607354

[25] Barzilai N, Huffman DM, Muzumdar RH, Bartke A. The critical role of metabolic pathways in aging. Diabetes. 2012;61(6):1315–22. 10.2337/db11-1300. 22618766 10.2337/db11-1300 PMC3357299

[26] Kenyon CJ. The genetics of ageing. Nature. 2010;464(7288):504–12. 10.1038/nature08980. 20336132 10.1038/nature08980

[27] Abbatecola AM, Lattanzio F, Molinari AM, Cioffi M, Mansi L, Rambaldi P, DiCioccio L, Cacciapuoti F, Canonico R, Paolisso G. Rosiglitazone and cognitive stability in older individuals with type 2 diabetes and mild cognitive impairment. Diabetes Care. 2010;33(8):1706–11. 10.2337/dc09-2030. 20435794 10.2337/dc09-2030 PMC2909046

[28] Narayan PJ, Lill C, Faull R, Curtis MA, Dragunow M. Increased acetyl and total histone levels in post-mortem Alzheimer’s disease brain. Neurobiol Dis. 2015;74:281–94. 10.1016/j.nbd.2014.11.023. 25484284 10.1016/j.nbd.2014.11.023

[29] Oh G, Ebrahimi S, Wang S-C, Cortese R, Kaminsky ZA, Gottesman II, Burke JR, Plassman BL, Petronis A. Epigenetic assimilation in the aging human brain. Genome Biology, 17(1). 2016; 10.1186/s13059-016-0946-8 10.1186/s13059-016-0946-8 PMC4848814 27122015

[30] Xie X, Hu H, Tong X, Li L, Liu X, Chen M, Yuan H, Xie X, Li Q, Zhang Y, Ouyang H, Wei M, Huang J, Liu P, Gan W, Liu Y, Xie A, Kuai X, Chirn G-W, Zhou H. The mTOR-S6K pathway links growth signalling to DNA damage response by targeting RNF168. Nature Cell Biology. 2018; 20(3):320–331. 10.1038/s41556-017-0033-8 10.1038/s41556-017-0033-8 PMC5826806 29403037

[31] Janeway CA. Immunobiology: the immune system in health and disease. New York: Garland Science; 2007.

[32] Wei W, Ji S. Cellular senescence: molecular mechanisms and pathogenicity. J Cell Physiol. 2018;233(12):9121–35. 30078211 10.1002/jcp.26956

[33] Davalli P, Mitic T, Caporali A, Lauriola A, D’Arca D. ROS, cell senescence, and novel molecular mechanisms in aging and age-related diseases. Oxid Med Cell Longev. 2016;2016:1–18. 10.1155/2016/3565127. 10.1155/2016/3565127 PMC4877482 27247702

[34] Miller KM, Tjeertes JV, Coates J, Legube G, Polo SE, Britton S, Jackson SP. Human HDAC1 and HDAC2 function in the DNA-damage response to promote DNA nonhomologous end-joining. Nat Struct Mol Biol. 2010;17(9):1144–51. 10.1038/nsmb.1899. 20802485 10.1038/nsmb.1899 PMC3018776

[35] Kinner A, Wu W, Staudt C, Iliakis G. H2AX in recognition and signaling of DNA double-strand breaks in the context of chromatin. Nucleic Acids Res. 2008;36(17):5678–94. 10.1093/nar/gkn550. 18772227 10.1093/nar/gkn550 PMC2553572

[36] Niedernhofer LJ, Kirkland JL, Ladiges W. Molecular pathology endpoints useful for aging studies. Ageing Res Rev. 2017;35:241–9. 10.1016/j.arr.2016.09.012. 27721062 10.1016/j.arr.2016.09.012 PMC5357461

[37] Brioschi S, Zhou Y, Colonna M. Brain parenchymal and extraparenchymal macrophages in development, homeostasis, and disease. J Immun. 2020;204(2):294–305. 10.4049/jimmunol.1900821. 31907272 10.4049/jimmunol.1900821 PMC7034672

[38] Alers S, Löffler AS, Wesselborg S, Stork B. Role of AMPK-mTOR-Ulk1/2 in the regulation of autophagy: cross talk, shortcuts, and feedbacks. Mol Cell Biol. 2012;32(1):2–11. 10.1128/mcb.06159-11. 22025673 10.1128/mcb.06159-11 PMC3255710

[39] Petersen RC. Mild cognitive impairment. N Engl J Med. 2011;364(23):2227–34. 10.1056/nejmcp0910237. 21651394 10.1056/nejmcp0910237

[40] Kaiser GS, Germann SM, Westergaard T, Lisby M. Phenylbutyrate inhibits homologous recombination induced by camptothecin and methyl methanesulfonate. Mutat Res: Fundam Mol Mech Mutagen. 2011;713(1–2):64–75. 10.1016/j.mrfmmm.2011.05.016. 10.1016/j.mrfmmm.2011.05.016 21658395

[41] Suto T, Karonitsch T. The immunobiology of mTOR in autoimmunity. J Autoimmun. 2020;110:102373. 10.1016/j.jaut.2019.102373. 31831256 10.1016/j.jaut.2019.102373

[42] Sadagurski M, Cady G, Miller RA. Anti-aging drugs reduce hypothalamic inflammation in a sex-specific manner. Aging Cell. 2017;16(4):652–60. 10.1111/acel.12590. 28544365 10.1111/acel.12590 PMC5506421

[43] Dai J, Jiang C, Chen H, Chai Y. Rapamycin attenuates high glucose-induced inflammation through modulation of mTOR/NF-κB pathways in macrophages. Front Pharmacol. 2019;10. 10.3389/fphar.2019.01292 10.3389/fphar.2019.01292 PMC6831745 31736762

[44] Hou H, Miao J, Cao R, Han M, Sun Y, Liu X, Guo L. Rapamycin ameliorates experimental autoimmune encephalomyelitis by suppressing the mTOR-STAT3 pathway. Neurochem Res. 2017;42(10):2831–40. 10.1007/s11064-017-2296-7. 28600752 10.1007/s11064-017-2296-7

[45] Liu T, Liang X, Sun Y, Yang S. Rapamycin suppresses the PI3K/AKT/mTOR signaling pathway by targeting SIRT1 in esophageal cancer. Exp Ther Med. 2021; 22(4). 10.3892/etm.2021.10624 10.3892/etm.2021.10624 PMC8406672 34475980

[46] Kojima H, Inoue T, Kunimoto H, Nakajima K. IL-6-STAT3 signaling and premature senescence. JAK-STAT. 2013;2(4):e25763. 10.4161/jkst.25763. 24416650 10.4161/jkst.25763 PMC3876432

[47] Sabe SA, Feng J, Sellke FW, Abid MR. Mechanisms and clinical implications of endothelium-dependent vasomotor dysfunction in coronary microvasculature. Am J Physiol Heart Circ Physiol. 2022;322(5):H819–41. 10.1152/ajpheart.00603.2021. 35333122 10.1152/ajpheart.00603.2021 PMC9018047

[48] Kim YC, Guan K-L. mTOR: a pharmacologic target for autophagy regulation. J Clin Invest. 2015;125(1):25–32. 10.1172/jci73939. 25654547 10.1172/jci73939 PMC4382265

